# Establishment of a 7-gene expression panel to improve the prognosis classification of gastric cancer patients

**DOI:** 10.3389/fgene.2023.1206609

**Published:** 2023-09-12

**Authors:** Mariana Belén Velásquez Sotomayor, Anthony Vladimir Campos Segura, Ricardo José Asurza Montalva, Obert Marín-Sánchez, Alexis Germán Murillo Carrasco, César Alexander Ortiz Rojas

**Affiliations:** ^1^ Immunology and Cancer Research Group (IMMUCA), Lima, Peru; ^2^ Escuela de Medicina Humana, Facultad de Ciencias de la Salud, Universidad Científica del Sur, Lima, Perú; ^3^ Biochemistry and Molecular Biology Research Laboratory, Faculty of Natural Sciences and Mathematics, Universidad Nacional Federico Villarreal, Lima, Peru; ^4^ Laboratory of Genomics and Molecular Biology, International Center of Research CIPE, A.C. Camargo Cancer Center, Sao Paulo, Brazil; ^5^ Departamento Académico de Microbiología Médica, Facultad de Medicina, Universidad Nacional Mayor de San Marcos, Lima, Peru; ^6^ Centro de Investigação Translacional em Oncologia (LIM24), Departamento de Radiologia e Oncologia, Faculdade de Medicina da Universidade de São Paulo and Instituto do Câncer do Estado de São Paulo, São Paulo, Brazil; ^7^ Laboratório de Investigação Médica (LIM) 31, Hospital das Clínicas HCFMUSP, Faculdade de Medicina, Universidade de São Paulo, São Paulo, Brazil

**Keywords:** prognosis, gastric cancer, score, risk classification, gene expression

## Abstract

Gastric cancer (GC) ranks fifth in incidence and fourth in mortality worldwide. The high death rate in patients with GC requires new biomarkers for improving survival estimation. In this study, we performed a transcriptome-based analysis of five publicly available cohorts to identify genes consistently associated with prognosis in GC. Based on the ROC curve, patients were categorized into high and low-expression groups for each gene using the best cutoff point. Genes associated with survival (AUC > 0.5; univariate and multivariate Cox regressions, *p* < 0.05) were used to model gene expression-based scores by weighted sum using the pooled Cox β regression coefficients. Cox regression (*p* < 0.05), AUC > 0.5, sensitivity > 0.5, and specificity > 0.5 were considered to identify the best scores. Gene set enrichment analysis (KEGG, REACTOME, and Gene Ontology databases), as well as microenvironment composition and stromal cell signatures prediction (CIBERSORT, EPIC, xCell, MCP-counter, and quanTIseq web tools) were performed. We found 11 genes related to GC survival in the five independent cohorts. Then, we modeled scores by calculating all possible combinations between these genes. Among the 2,047 scores, we identified a panel based on the expression of seven genes. It was named GES7 and is composed of *CCDC91*, *DYNC1I1*, *FAM83D*, *LBH*, *SLITRK5*, *WTIP*, and *NAP1L3* genes. GES7 features were validated in two independent external cohorts. Next, GES7 was found to recategorize patients from AJCC TNM stages into a best-fitted prognostic group. The GES7 was associated with activation of the TGF-β pathway and repression of anticancer immune cells. Finally, we compared the GES7 with 30 previous proposed scores, finding that GES7 is one of the most robust scores. As a result, the GES7 is a reliable gene-expression-based signature to improve the prognosis estimation in GC.

## 1 Introduction

Gastric cancer (GC) is responsible for over one million new cases in 2020 and an estimated 769,000 deaths, ranking fifth for incidence and fourth for mortality globally (Sung et al., 2021). Gastric adenocarcinoma is the more common subtype, comprising more than 95% of cases, and is a highly heterogeneous group concerning anatomical location, histological subtypes, and molecular features (Kupfer, 2017; Yan et al., 2022). Although a variety of new cancer treatments have been introduced that have led to improvement of life expectancy in GC, the standard treatment continues to be surgical resection, chemotherapy, and/or radiotherapy, with five-year survival rates from 90% to 98% for pT1 stage tumors and from 38% to 59% for pT2–4 tumors (Yan et al., 2022).

High-throughput sequencing efforts have been made to characterize the genomic landscape of gastric cancer, allowing better categorization of the heterogeneity among patients. The Cancer Genome Atlas (TCGA) developed a robust molecular classification and identified dysregulated pathways and candidate drivers of distinct classes of gastric cancer (Bass et al., 2014). TCGA classification divides GC into four subtypes: tumors positive for Epstein–Barr virus (EBV); microsatellite instability (MSI); genomically stable tumors (GS); and tumors with chromosomal instability (CIN) (Bass et al., 2014). Similarly, the Asian Cancer Research Group (ACRG) established clinically relevant molecular subtypes based on gene expression analysis and mutations (Cristescu et al., 2015). ACRG classification describes four molecular subtypes linked to disease progression and prognosis: microsatellite instability (MSI), microsatellite instability (MSS)/Epithelial-Mesenchymal Transition (EMT), MSS/TP53 mutated, and MSS/TP53 wild-type (Cristescu et al., 2015).

Although these classifications could be valuable for clinical decisions, the American Joint Committee on Cancer (AJCC) TNM staging (Liu et al., 2018) remains the primary prognostic determinant in GC. Since the disease can sometimes progress unexpectedly to what is established by the TNM classification, the clinical application of biological and molecular markers is pending as a complement to AJCC TNM staging to improve the prognosis. A possible reason for this pending is the need for more consensus on how efficiently to include in the laboratory routine the determination of *TP53* mutations (Malcikova et al., 2018), CIN (McGranahan et al., 2012), and EMT (Cristescu et al., 2015).

Some studies have proposed gastric tumor biomarkers from tissue or blood samples of GC patients through different analytic strategies (Wang et al., 2016; Wang et al., 2017; Cheong et al., 2018; Shi et al., 2018; Zhu et al., 2020; Li et al., 2021) with clinically relevant results. Although their relevant contribution to the field, some of these panels face some limitations, like the high number of component genes, the low number of cohorts evaluated (generally 1–3 datasets), and the geographic origin of these cohorts. In the present study, we aim to improve the current GC biomarker context by using a robust algorithm to evaluate all available genes in over one thousand samples from five different cohorts. After detecting the most relevant genes for assessing the prognosis of GC patients in the five cohorts, we performed mathematical modeling to get a better gene-expression panel. Thus, we found a score based on the expression of seven genes, herein referred to as GES7. This score was validated in two independent cohorts and demonstrated to be robust in comparison with previous proposed scores/indexes.

## 2 Materials and methods

### 2.1 Patient cohorts and gene expression profiling

This study analyzed publicly available clinical and transcriptomic data of five GC cohorts, as discovery datasets. First, The Cancer Genome Atlas (TCGA) gastric cancer cohort (*n* = 345) (Bass et al., 2014) with RNA-seq data was included (Table 1). Transcriptome data of the TCGA cohort were generated using HiSeq 2000 (Illumina) and included information on 20,508 genes. Clinical and genetic information were retrieved from the Firebrowse data portal site (http://firebrowse.org/). Next, four transcriptome datasets based on the microarray were included: Asian Cancer Research Group (ACRG, GSE66229 dataset, *n* = 297) (Cristescu et al., 2015), Yonsei University Severance Hospital (YUSH, GSE84437 dataset, *n* = 433) (Kim et al., 2019), Korea University Guro Hospital (KUGH, GSE26899 data set, *n* = 93) (Oh et al., 2018) and the National Cancer Centre of Singapore (NCCS, GSE15459 data set, *n* = 192) (Ooi et al., 2009) (Table 1). Microarray data were retrieved from the Gene Expression Omnibus (GEO) database (www.ncbi.nlm.nih.gov/geo/). Data from ACRG and NCCS cohorts were generated using Human Genome U133 Plus 2.0 (Affymetrix) which contained probes for 23,520 genes. In comparison, YUSH and KUGH cohorts were generated using HumanHT-12 V3.0 (Illumina) which contained probes for 25,124 genes. As external and independent validation datasets, we used additional tumor specimens collected from the Kosin University College of Medicine (KUCM, GSE26901 dataset, *n* = 109) and The University of Texas MD Anderson Cancer Center (MDACC, GSE28541 data set, *n* = 40), described in a previous study (Oh et al., 2018).

**TABLE 1:** Clinical and biological information of gastric cancer cohorts.

Level		TCGA	ACRG	YUSH	KUGH	NCCS
n		345	297	433	93	192
Age [median (IQR)]		67.00 [58.00, 72.00]	64.00 [55.00, 70.00]	62.00 [53.00, 68.00]	60.00 [50.00, 69.00]	66.55 [56.80, 73.00]
Sex, n (%)	Female	122 (35.4)	101 (34.0)	137 (31.6)	20 (21.5)	67 (34.9)
	Male	223 (64.6)	196 (66.0)	296 (68.4)	73 (78.5)	125 (65.1)
Race category, n (%)	Asian	73 (21.2)	—	—	—	—
	Black or African American	9 (2.6)	—	—	—	—
	White	218 (63.2)	—	—	—	—
	Unknown	45 (13)	—	—	—	—
Histologic grade, n (%)	G1	6 (1.7)	—	—	—	—
	G2	116 (33.6)	—	—	—	—
	G3	215 (62.3)	—	—	—	—
	GX	8 (2.3)	—	—	—	—
Tumor weight (median [IQR])		300.00 [135.50, 821.50]	—	—	—	—
Tumor stage T, n (%)	T1/T2	88 (25.5)	185 (62.3)	49 (11.3)	—	—
	T3/T4	257 (74.5)	112 (37.7)	384 (88.7)	—	—
Nodal status, n (%)	N0	107 (31.0)	38 (12.8)	80 (18.5)	—	—
	N1/N2/N3	238 (69.0)	259 (87.2)	353 (81.5)	—	—
Metastasis status, n (%)	M0	320 (92.8)	270 (90.9)	—	85 (92.4)	—
	M1	25 (7.2)	27 (9.1)	—	7 (7.6)	—
AJCC TNM staging, n (%)	I	—	—	—	11 (12.0)	31 (16.1)
	IA	9 (2.6)	0 (0)		—	—
	IB	3 (0.9)	31 (10.4)	—	—	—
	II	54 (15.7)	94 (31.6)	—	18 (19.6)	29 (15.1)
	III	—	—	—	27 (29.3)	72 (37.5)
	IIIA	188 (54.5)	69 (23.2)	—	—	—
	IIIB	31 (9.0)	26 (8.8)	—	—	—
	IV	60 (17.4)	77 (25.9)	—	36 (39.1)	60 (31.2)
Lauren classification, n (%)	Intestinal	152 (44.1)	144 (48.5)	—	59 (64.1)	75 (39.1)
	Diffuse	73 (21.2)	134 (45.1)	—	31 (33.7)	99 (51.6)
	Mixed	—	19 (6.4)	—	2 (2.2)	18 (9.4)
	Unknown	120 (34.8)	—	—	—	—
Primary tumor site, n (%)	Fundus/Body	123 (35.7)	—	—	31 (33.3)	—
	Cardia/Proximal	45 (13.0)	—	—	7 (7.5)	—
	Antrum/Distal	131 (38.0)	—	—	55 (59.1)	—
	Gastroesophageal junction	35 (10.1)	—	—	—	—
	Unknown	11 (3.2)	—	—	—	—
	Body	—	107 (36.0)	—	—	—
	Cardia or cardia + body	—	31 (10.4)	—	—	—
	Antrum	—	149 (50.2)	—	—	—
	Body + antrum or fundus to antrum	—	6 (2.0)	—	—	—
	Entire stomach	—	4 (1.3)	—	—	—
TCGA molecular subtype, n (%)	MSI	66 (19.1)	—	—	—	—
	EBV	27 (7.8)	—	—	—	—
	CIN	198 (57.4)	—	—	—	—
	POLE	7 (2.0)	—	—	—	—
	GS	47 (13.6)	—	—	—	—
ACRG molecular subtype, n (%)	MSI	—	68 (22.9)	—	—	—
	TP53 mut	—	77 (25.9)	—	—	—
	TP53 wt	—	106 (35.7)	—	—	—
	EMT	—	46 (15.5)	—	—	—
NCCS molecular subtype, n (%)	Invasive	—	—	—	—	51 (26.6)
	Metabolic	—	—	—	—	40 (20.8)
	Proliferative	—	—	—	—	70 (36.5)
	Unstable	—	—	—	—	31 (16.1)

### 2.2 Identification of genes associated with prognosis

After collecting gene expression data, we used survival receiver operating characteristic (ROC) curves to calculate the optimal gene expression cutoff value for each gene stratifying patients into high and low-expression level groups. About the sample size of high/low expression groups, we considered a cutoff value to preserve at least 20% of the total sample size in these groups avoiding possible overfitting, groups with small sample sizes, and biased selection of cutoff value. If multiple cutoffs were retrieved for each gene, the cutoff value closer to the median was selected. Overall survival (OS) events were used in this calculation. Then, these groups were compared by univariate and multivariate Cox proportional hazard regression for each gene in each cohort. Possible confounding variables were included in the multivariate analysis depending on data availability. Thus, we adjusted prognosis prediction with the following potential confounders: age (as continuous), sex, Lauren classification, T stage, N and M status, primary tumor site, and molecular subtype. We considered that a gene is associated with prognosis if the area under the survival ROC curve (AUC) was >0.5, and a *p*-value < 0.05 for Cox regressions, in the five cohorts in an independent analysis for each cohort.

### 2.3 Modeling of gene-expression-based index

To improve the prognosis prediction of the gene signatures, we modeled gene expression-based scores using the genes identified as described in the previous section. The scores were calculated by the weighted sum of gene expression values, using the β coefficient of Cox regression, according to the next formula:

$$Score={Gene}_{1}\beta_{1}+{Gene}_{2}\beta_{2}+{Gene}_{3}\beta_{3}+...+{Gene}_{i}\beta_{i}$$

The β value corresponds to pooled β coefficient calculated from the five cohorts by gene and estimated by a fixed- and random-effects model according to the heterogeneity between studies. Heterogeneity was assessed using Higgin’s I2 statistic and Cochran’s Q-test (Higgins and Thompson, 2002). A score was considered highly predictive when univariate HR > 2 (*p* < 0.05), multivariate HR > 1.5 (*p* < 0.05), AUC > 0.5, sensitivity > 0.5, and specificity > 0.5, in the five cohorts in an independent analysis for each cohort. Then, we selected the best gene expression-based score after considering the HR values and the number of genes constituting the panel. Finally, to homogeneously apply the score to any cohort, we normalized the score by dividing the score values by the median of the score calculated for each discovery dataset. Then, we established three groups according to the score values: low (score < 0.9), intermediate (0.9 ≤ score < 1.1), and high (score ≥ 1.1) groups.

### 2.4 Biological pathways enrichment

We performed the Gene Set Enrichment Analysis (GSEA) using the Broad Institute software (http://software.broadinstitute.org/gsea/index.jsp) to find biological processes associated with the score. Gene Ontology (GO), Kyoto Encyclopedia of Genes and Genomes (KEGG), and Reactome databases were included in our analysis. Enrichment scores were calculated based on Kolmogorov-Smirnov statistics tested for significance using 1,000 permutations. Additionally, Pearson correlation was used as a metric for ranking genes. A pathway was considered enriched when a nominal *p*-value and FDR q-value were <0.05.

### 2.5 Enrichment of cells signatures and drug sensitivity evaluation

The immune stromal cell signature of tumor samples was evaluated by using the following algorithms: CIBERSORT (Newman et al., 2015), EPIC (Racle and Gfeller, 2020), xCell (Aran et al., 2017), MCP-counter (Becht et al., 2016), and quanTIseq (Finotello et al., 2019); from the TIMER web tool (http://timer.cistrome.org/). Next, we explored the drug response in gastric cancer cells with different score values. The gene expression score was calculated in the following gastric cancer cell lines: AGS, FU97, GCIY, HGC-27, Hs-746T, IM-95, MKN45, MKN7, NCI-N87, NUGC-3, RERF-GC-1B, SNU-1, SNU-16, SNU-5, TGBC11TKB, and 23132-87. Gene expression data were retrieved from the Expression Atlas database (www.ebi.ac.UK/gxa/experiments/E-MTAB-2770/). Then, we correlate the scores with the inhibitory concentration 50 (IC50) for 448 drugs (https://www.cancerrxgene.org/). Spearman rho coefficients >0.25 or < −0.25 with *p* < 0.05 were considered to identify drug responses.

### 2.6 Statistical analysis

The OS was defined as the time from diagnosis to death for any cause; those alive or lost to follow-up were censored. Relapse-free survival (RFS) or disease-free survival (DFS) was defined as the time from the achievement of response to the first adverse event. That is relapse or death from any cause, whichever occurs first. Kaplan-Meier (KM) plots were performed to demonstrate the power of stratification. The Mann-Whitney test and Fisher’s exact test were used to compare clinical and biological characteristics between patients. All *p*-values were two-sided with a significance level of 0.05. All calculations were performed using R 4.1.1 (The CRAN project, www.r-project.org) software.

## 3 Results

### 3.1 Survival-related genes identified a 7-gene expression-based score applied to risk stratification in gastric cancer

**FIGURE 1:**
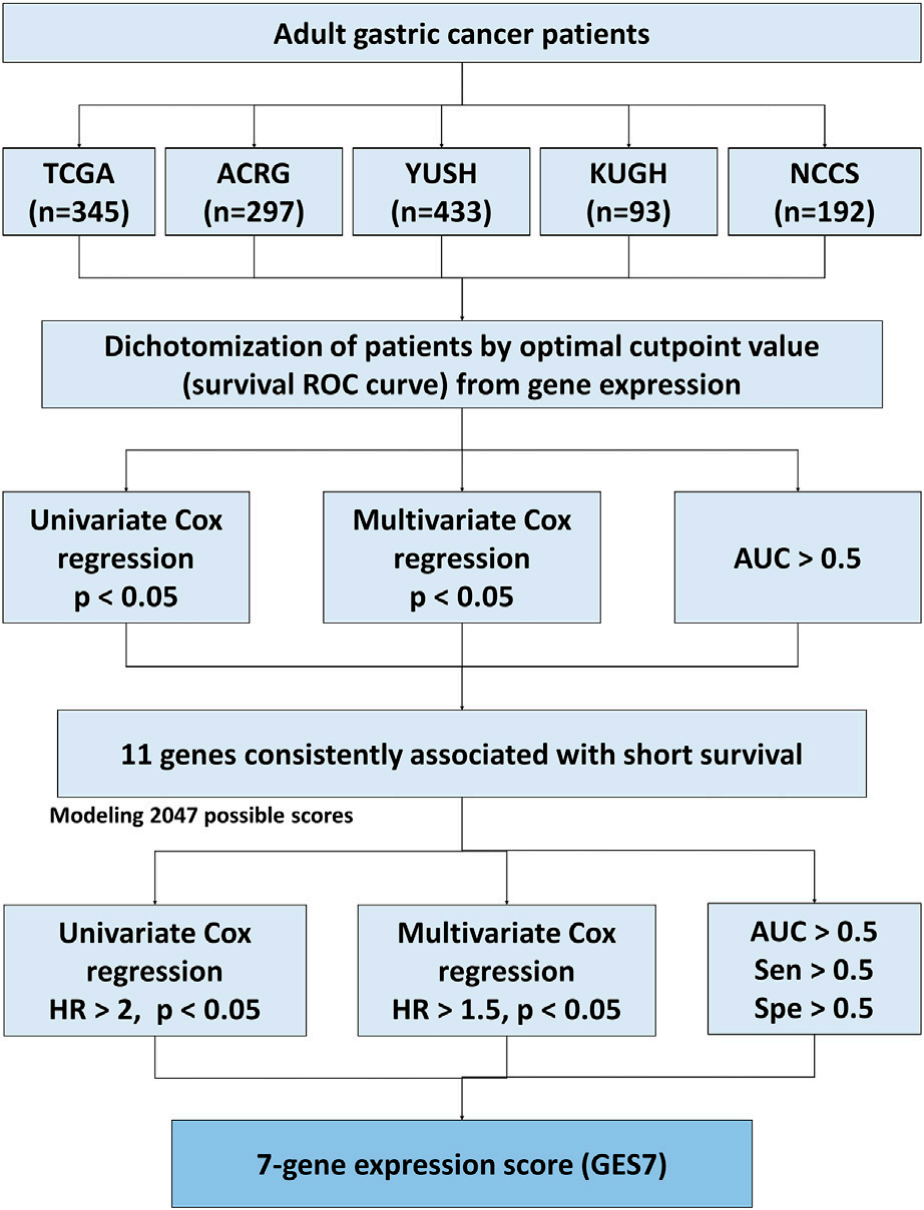
Flow diagram of the score construction based on gene expression for GC.

**FIGURE 2:**
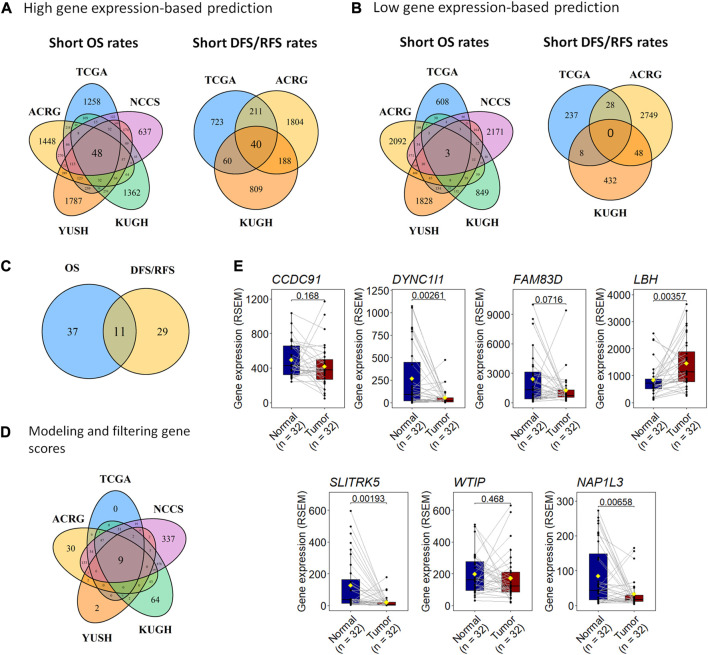
A 7-gene expression score, GES7, can predict prognosis in gastric cancer. **(A)** We used five transcriptome datasets to find 48 genes with high expression associated with unfavorable OS and 40 genes related to unfavorable DFS (TCGA and ACRG) or RFS (KUGH). **(B)** Also, the high expression of three genes was associated with favorable OS, but neither predicts DFS (TCGA and ACRG) or RFS (KUGH). **(C)** By merging these results, 11 genes were associated with prognosis in all datasets. **(D)** Nine scores based on gene expression were highly predictive of prognosis, where a 7-gene expression panel (GES7) was the most parsimonious. **(E)** Comparison of gene expression in tumor versus healthy tissues of genes composing GES7.

To identify survival-related genes in gastric cancer (GC), we analyzed the transcriptome data of five GC cohorts (Table 1). We dichotomized cohorts according to gene expression using the survival ROC curve. Then, for prognosis evaluation, high and low expression categories were established for each gene in each cohort (Figure 1). We identified 48 genes whose high expression was associated with short overall survival rates. Furthermore, 40 genes were consistently associated with disease-free and relapse-free survival in TCGA, ACRG, and KUGH cohorts (Figure 2A). On the other hand, low expression of three genes was associated with short OS rates, although none of these could predict DFS or RFS (Figure 2B); consequently, these genes were not considered in further analysis. By merging these results, we identified 11 genes associated with prognosis (Figure 2C). Supplementary Tables S1–S5 show the association between gene expression and overall survival for all genes included in the platforms for each cohort. Next, based on the 11 genes we found, we modeled 2,047 gene expression scores, which refer to all unique combinations among these 11 genes. To uniformize our score calculations, we pooled the β coefficients of the five cohorts for each gene (Supplementary Figures S1–S3). After applying Cox regression and AUC discrimination, 9 scores showed an improved power of survival prediction in the five cohorts (Figure 2D; Supplementary Figure S4). Next, we selected the most parsimonious score, based on seven genes, here referred to as GES7. The GES7 is composed of *CCDC91*, *DYNC1I1*, *FAM83D*, *LBH*, *SLITRK5*, *WTIP*, and *NAP1L3*. Expression levels of these genes and the comparisons with their normal counterparts are shown in Figure 2E. Also, Kaplan-Meier plots showing the stratification power of these genes are shown in Supplementary Figures S5–S8 (Log-rank, *p* < 0.05).

**FIGURE 3:**
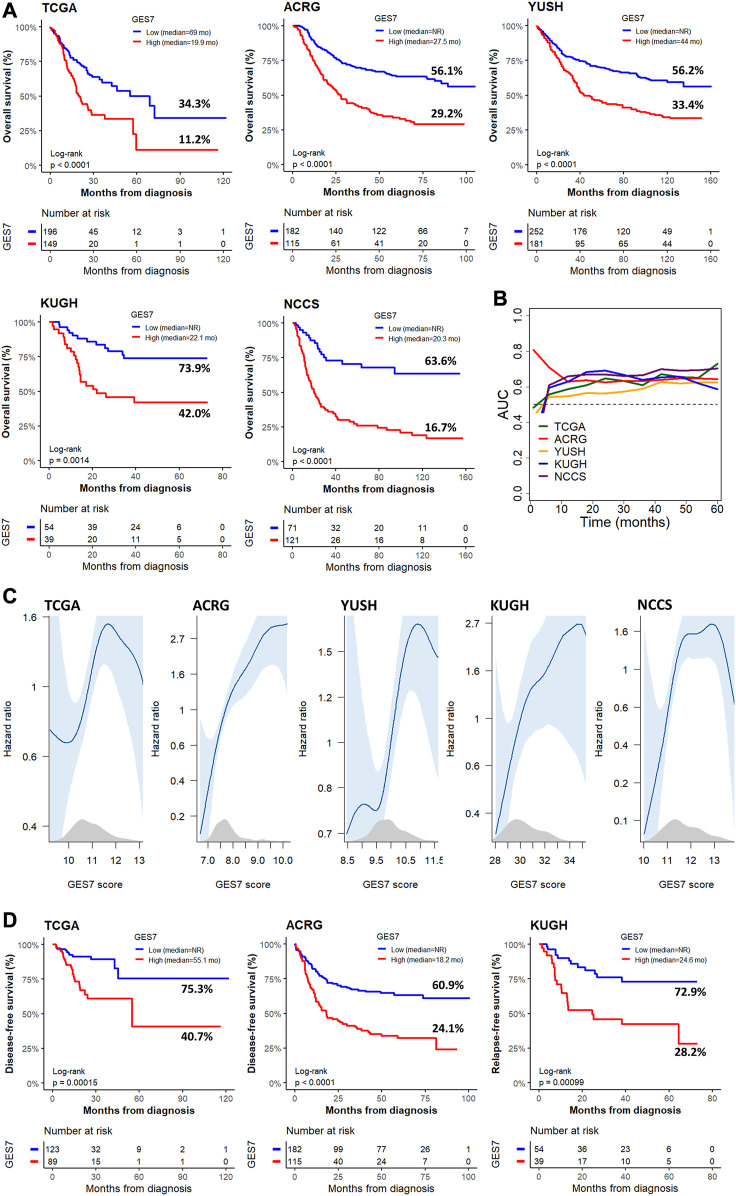
Survival of GC patients according to the GES7 values. **(A)** Kaplan-Meier plots show the survival stratification power of GES7 for OS. **(B)** Survival AUC analysis of GES7 until 5 years of follow-up. **(C)** Smooth plot showing the relation between the risk of death and the GES7 values. **(D)** Kaplan-Meier plots show the survival stratification power of GES7 for DFS and RFS.

**FIGURE 4:**
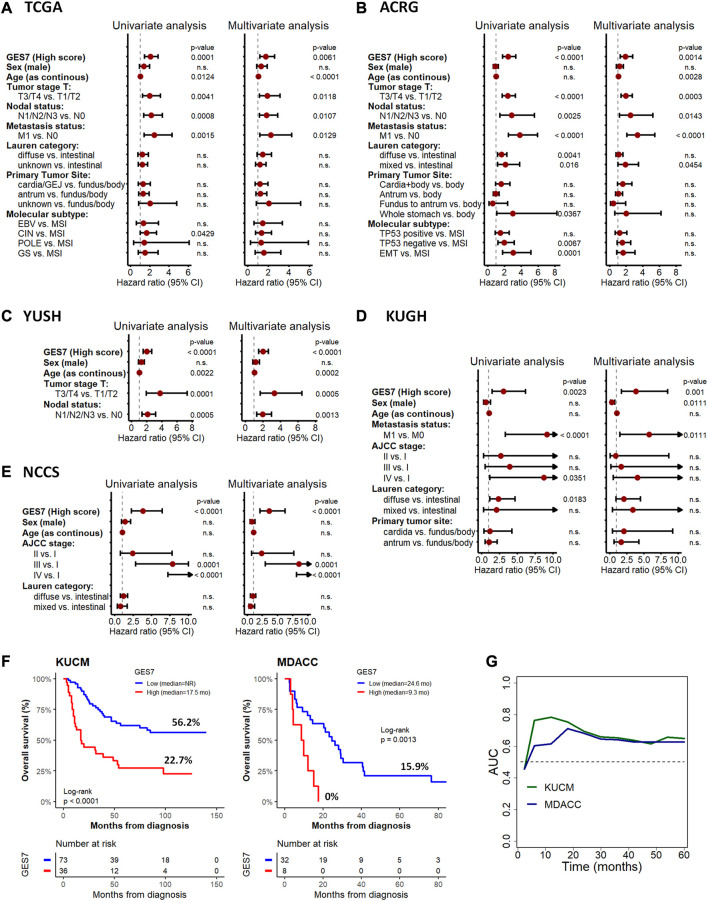
Cox regression for overall survival according to GES7 and validation analysis. Univariate and multivariate analyses were performed for the TCGA **(A)**, ACRG **(B)**, YUSH **(C)**, KUGH **(D)**, and NCCS **(E)** cohorts, showing that GES7 is an independent prognostic factor in gastric cancer. Also, Kaplan-Meier plots **(F)** and ROC curve analysis **(G)** in KUCM and MDACC validation datasets confirm the clinical applicability of GES7.

The GES7 was highly efficient in stratifying overall survival in GC patients (Figure 3A). Thus, GES7^high^ patients had inferior survival rates, with median OS of 19.9 vs. 69 months for TCGA, 27.5 months vs. not reached for ACRG, 44 months vs. not reached for YUSH, 22.1 months vs. not reached for KUGH, and 20.3 months vs. not reached for NCCS. In all cases, the AUC values of the GES7^high^ patients were higher than 0.5 (Figure 3B). Consistent with these results, we found that the GES7 score covariate with pointwise estimates of the overall survival hazard ratios (HRs) (Figure 3C). Also, GES7^high^ patients were more likely to relapse according to the disease- and relapse-free survival analysis (Figure 3D). Furthermore, after including potential confounding variables in our Cox regression analysis, GES7 remained as a relevant prognostic marker with HRs of 1.75 (*p* = 0.0061), 1.88 (*p* = 0.0014), 2.01 (*p* < 0.0001), 3.8 (*p* = 0.001), and 3.59 (*p* < 0.0001), for TCGA, ACRG, YUSH, KUGH, and NCCS, respectively (Figures 4A–E). Although GES7^low^ patients included more MSI cases and GES7^high^ patients showed more CIN, GS, and EMT cases (Supplementary Tables S6, S7), our multivariate regression demonstrates that the molecular subtypes are not a confounder variable. Interestingly, we found that GES7^high^ was associated with an invasive phenotype as described for the NCCS cohort (Supplementary Table 10). No other clinical variable was consistently associated with the GES7 (Supplementary Tables S6–S10). Finally, we validated our GES7 in two independent cohorts, KUCM and MDACC. We found that GES7^high^ patients had inferior survival rates, with median OS of 17.5 months vs. not reached (log-rank *p* < 0.0001), and 9.3 vs. 24.6 months (log-rank *p* = 0.0013), respectively (Figure 4F). Also, ROC curve analysis demonstrated that the area under the curve values remained consistently high, exceeding 0.6, for up to 5 years of follow-up in both validation cohorts (Figure 4G). Therefore, the GES7 gene signature proved to be highly efficient in stratifying overall survival in gastric cancer patients across multiple cohorts.

### 3.2 GES7 is a promissory tool for recategorizing AJCC TNM subgroups

Next, we combined the traditional anatomical staging system with the molecular signature of GES7. For this purpose, we normalized the GES7 values across the five cohorts to find widely applicable cutoffs despite the techniques used to measure gene expression (RNA-seq or microarray) (Supplementary Figure S9). Next, we established three categories, GES7 low, intermediate, and high, applying cut-off points of 0.9 and 1.1 for all cohorts. By considering these categories, we proposed a risk classification system that integrated the AJCC TNM staging and the GES7 (Supplementary Table 11). As seen in Supplementary Figure 10A, the three categories of GES7 identified patients with different survival rates in the ACRG cohort. As expected, the AJCC TNM staging (7th edition) also efficiently distributed patients into other prognostic groups (Supplementary Figure 10B). After integrating both systems, we observed a reclassification of patients in a best-fitted risk group (Supplementary Figures 10C–D). Considering the IIIA category as intermediate for prognosis (median OS_IIIA_ = 68.1 months) and IIIB and IV categories as adverse (median OS_IIIB_ = 28.2 months and median OS_IV_ = 17.4 months), we had that the GES7 restructured these groups by the formation of an adverse-risk group constituting of patients from IIIA, IIIB and IV (median OS_adverse_ = 25.2 months), leaving the remaining patients of IV group forming the very-adverse risk group (median OS_adverse_ = 12.7 months) (Supplementary Figures 10D, E). These results were validated in the TCGA cohort, where the adverse and very-adverse risk groups differed from the intermediate and favorable groups compared to the AJCC TNM system alone (Supplementary Figures 11A–E). Therefore, the integration of our gene signature with the classic anatomical staging system improves prognostication accuracy and provides clinicians with a more comprehensive assessment of the patient’s disease.

### 3.3 GES7 predicts stromal cells-enriched microenvironment

**FIGURE 5:**
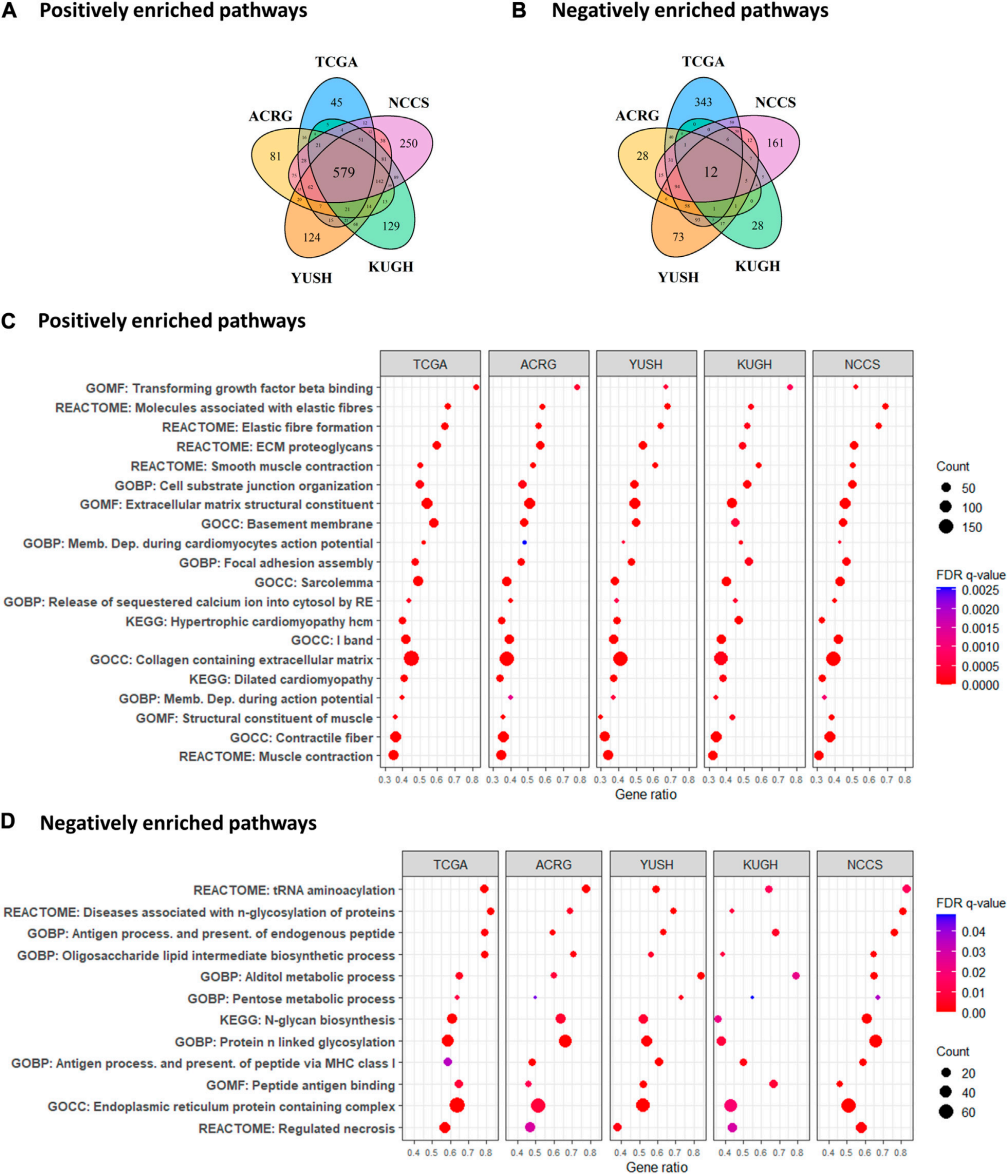
Enrichment analysis of biological pathways according to GES7. **(A)** Number of positive and **(B)** negative correlated paths with GES7. **(C)** Gene set enrichment analysis demonstrated an invasive and motility phenotype in tumors with high GES7, while **(D)** antigen-presenting processes were diminished.

**FIGURE 6:**
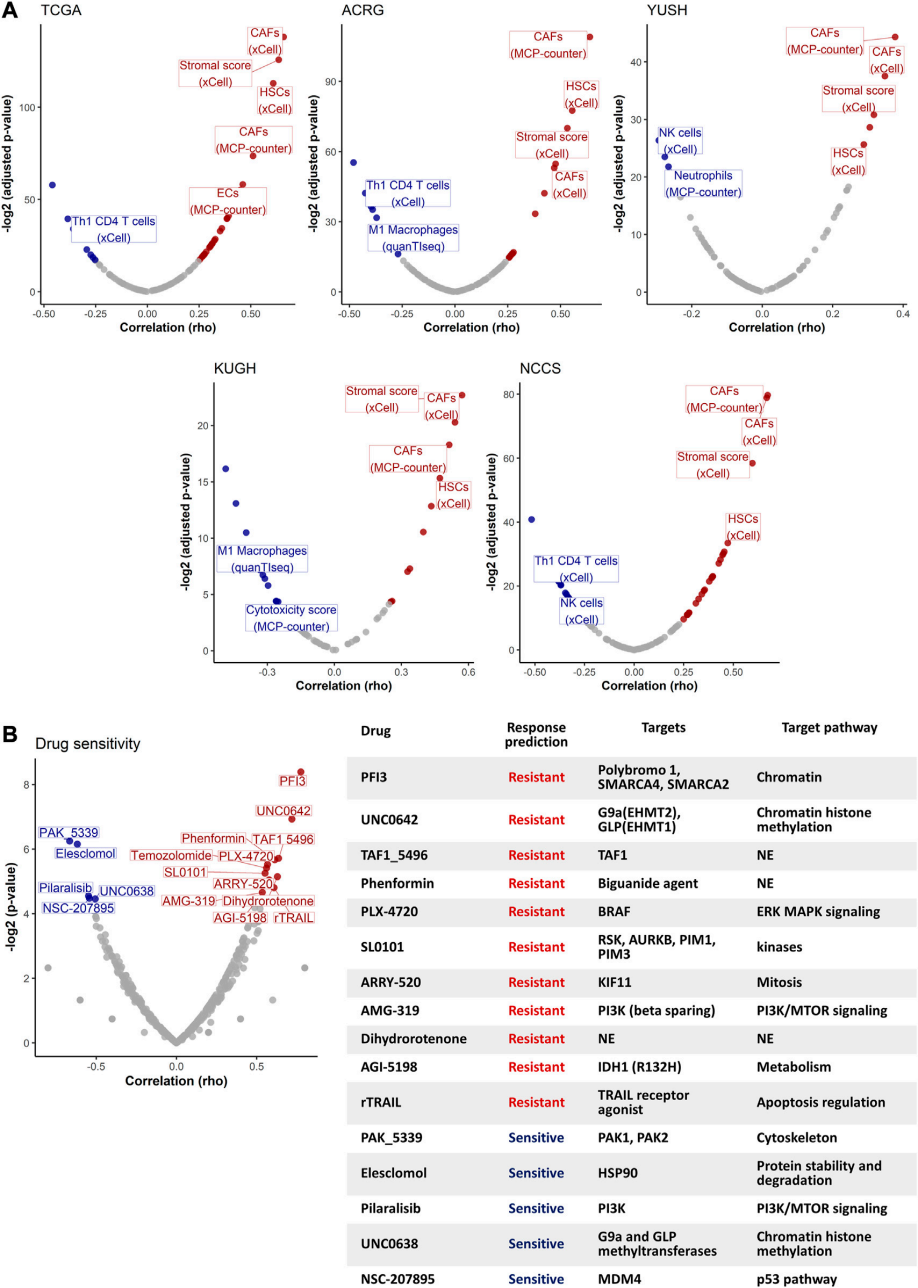
Non-tumor cell type abundance and drug sensitivity analysis. **(A)** Volcano plots show the correlation between GES7 values and non-tumor cell type abundance. **(B)** Drug sensitivity analysis of GC cell lines. The volcano plot (left) shows the correlation between the GES7 and the IC50 for each drug. The table (right) shows the description for each drug that was correlated with GES7.

To explore GES7-related biological pathways, we performed a GSEA with the transcriptome from the five discovery datasets. Figures 5A, B show that 579 pathways were consistently enriched in GES7 high samples, while 12 were for GES7^low^. The top enriched pathway was the transforming growth factor beta (TGF-β) activation (Figure 5C). In line with this result, pathways related to elastic fiber were enriched, indicating a motility phenotype. On the other hand, antigen processing and presentation-related pathways were negatively enriched, indicating low activity of the innate immune system in these tumors (Figure 5D). Then, we tested the hypothesis of non-tumor cell infiltrations being associated with GES7. We found that GES7^high^ tumors are enriched with stromal cells like cancer-associated fibroblasts and have a reduced presence of innate immune cells (Figure 6A). Finally, we found that GC cell lines with high GES7 respond well to *in vitro* treatment with PAK_5339. This drug inhibits the p21-activated kinases 1 (PAK1) and 2 (PAK2), Elesclomol (anti-HSP90), Pilaralisib (anti-PI3K), UNC0628 (anti-G9a and GLP methyltransferases), and NSC-207895 (anti-MDM4) (Figure 6B), that could be valuable treatment strategies for GES7^high^ patients.

### 3.4 Comparison of scores/indexes reveals GES7 as a robust biomarker of prognosis

**FIGURE 7:**
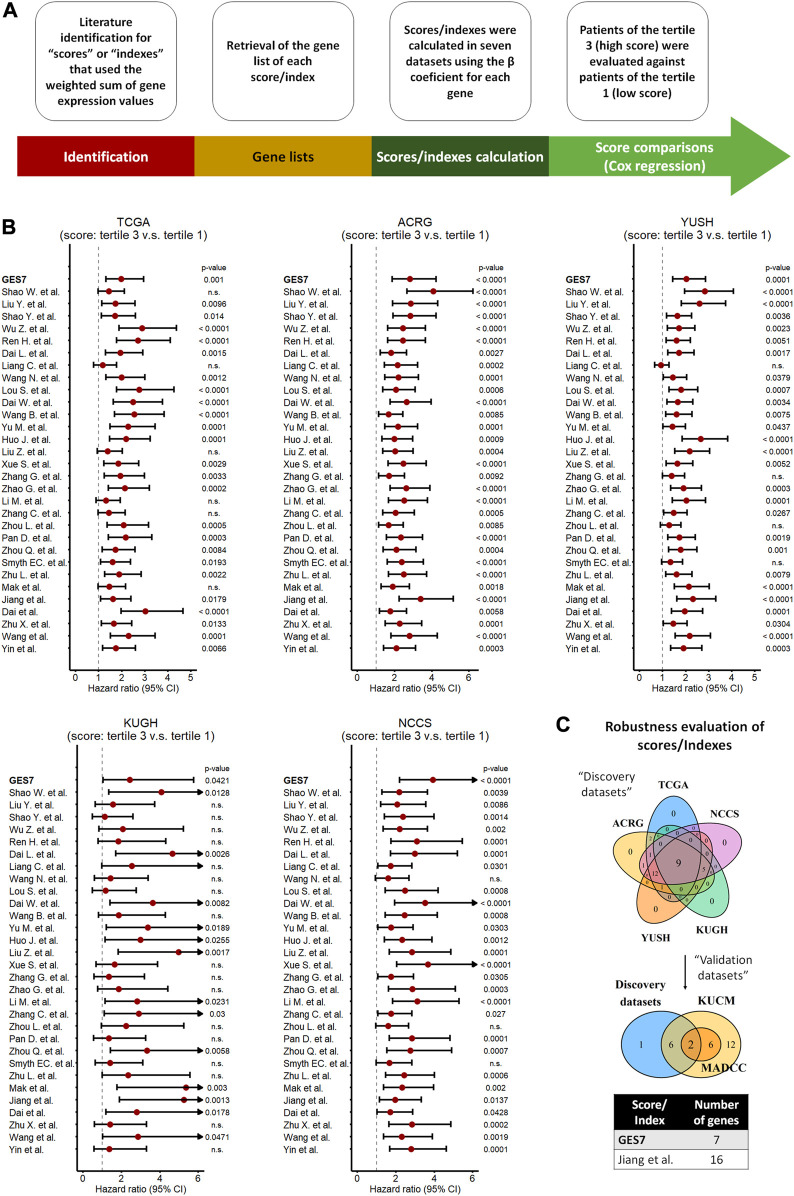
Comparison of scores/indexes based on gene expression to evaluate prognosis in GC. **(A)** Strategy used to calculate and apply the scores to our datasets. **(B)** Univariate Cox regression analysis of each score in our discovery datasets. **(C)** Merged results between discovery and validation datasets indicating the most robust scores, which included the GES7.

Previous gene expression-based prognostic indexes have been proposed as predictors of survival in GC. Here, we compared the GES7 with 30 prognostic scores published between 2013 and 2023 (Supplementary Table 12). After retrieving the gene list of each score, we calculated them using the weighted sum of gene expression values, the base strategy of all the studies. To homogenize the comparison between scores, we divided the cohorts into tertiles, and considered tertile 3 as the high score group, while tertile 1 as the low score group (Figure 7A). After applying the scores to our discovery datasets by univariate Cox regression analysis (Figure 7B), we found that 9 of 31 indexes were able to discriminate between patients of different risk groups. Finally, when we added the evaluation of our validation datasets, only two scores, within which is the GES7, were shown as the robust indexes (Figure 7C; Supplementary Figure 12).

## 4 Discussion

Gene expression signatures are a valuable tool for risk categorization in GC (Chen et al., 2005; Shi et al., 2018). In this study, our primary objective was to develop a robust and interpretable genetic index for predicting prognosis in gastric cancer. We opted for a methodology that is straightforward, easy to interpret, and based on widely accepted statistical techniques. We utilized survival ROC curves to determine the optimal gene expression cutoff value for each gene, which allows us to dichotomize patients into high and low-expression level groups based on prognostic relevance. Furthermore, we performed univariate and multivariate Cox proportional hazard regression analyses to identify genes significantly associated with prognosis in each cohort while adjusting for potential confounders. The multivariate analysis allowed us to consider the influence of several relevant clinical variables, enhancing the clinical applicability of our findings. Regarding the modeling of gene expression-based scores, we calculated the scores using the weighted sum of gene expression values based on the β coefficient from Cox regression. This approach allows us to assign appropriate weights to individual genes based on their prognostic significance, making the index a reflection of gene contributions to overall survival outcomes. As result of this process, a gene expression signature based on seven genes (GES7) was established. For four of them, *WTIP*, *NAP1L3*, *CCDC91*, and *SLITRK5*, this is the first study in which an association between their expression levels and the prognosis of GC was found. Interestingly, studies in other cancers had already identified *WTIP*, *NAP1L3*, and *CCDC91*, as relevant genes for tumor progression (Zeng et al., 2016; Heshmati et al., 2018; Wu et al., 2019; Lv et al., 2021). In contrast, mutations in *SLITRK5* have been described as predictors of prolonged survival in GC patients with *TP53* wild-type status (Park et al., 2016). In contrast, the high expression of *DYNC1I1*, *FAM83D*, and *LBH*, have been previously associated with poor prognosis and an aggressive tumor phenotype in GC (Huang et al., 2017; Deng et al., 2018; Chonov et al., 2019; Gong et al., 2019; Yu et al., 2019; Zhang et al., 2019).

Considering the expression status of these seven genes, we found that GES7^high^ tumors are characterized by the activation of the transforming growth factor beta (TGF-β), an essential fibrogenic agent that can promote an invasive phenotype, immunosuppression, and an interplay between tumor and stromal cells (Balkwill et al., 2012; Colak and ten Dijke, 2017; Henke et al., 2020). These results align with the association between GES7^high^ and the high number of EMT cases (ACRG dataset) or invasive (NCCS) subtypes. Also, a high stromal cell abundance was associated with GES7^high^ after applying CIBERSORT-based algorithms. In addition, the endogenous antigen presentation process pathway, M1-macrophages, and Th1 CD4 T cell abundance were negatively correlated with the GES7. Usually, antigens derived from tumor-specific mutations should be displayed on the surface of cells by MHC-I, processed by cells like macrophages, and presented to T lymphocytes. However, tumor cells can exploit multiple escape mechanisms to evade immune recognition (Jhunjhunwala et al., 2021). Thus, our GES7 score has the potential to identify tumors that evade the immune system. Other suppressed pathways in GES7^high^ tumors were the DNA replication and cell cycle. The suppression of DNA repair induces an accumulation of somatic mutation, which is usually related to a worse prognosis (Turgeon et al., 2018), consistent with our results.

Next, we found that GES7^high^ GC cells could respond to treatment with PAK_5339, Elesclomol, Pilaralisib, UNC0638, and NSC-207895. Among therapeutic proposals with current tests in cancer patients, we have PAK_5339, which is a drug that inhibits PAK1 and PAK2, proteins related to the activation of cell survival by different mechanisms, such as the metabolism of Bcl-2 in the mitochondria, the modulation of the cell motility, and the activation of the PI3K-AKT/mTOR pathway (Yao et al., 2020; Liu et al., 2021). Consequently, these PAK-promoted pathways could be targets of putative drugs for treating GES7^high^ patients. On the other hand, Elesclomol, an inducer of oxidative stress (Qu et al., 2009), and Pilaralisib, an inhibitor of PI3K (Hashemzadeh et al., 2018), have been tested in combination with conventional treatments in other types of cancers (Qu et al., 2009; Tolaney et al., 2014; Bechter et al., 2016; Hashemzadeh et al., 2018; Buccarelli et al., 2021). Therefore, our results and the previous evaluation of these drugs in brain and breast tumors warrant for further experiments.

Several previous gene expression-based studies have proposed biomarkers for GC (Szász et al., 2016; Wang et al., 2016; Wang et al., 2017; Cheong et al., 2018; Shi et al., 2018; Li et al., 2020; Liu et al., 2020; Zhu et al., 2020; Li et al., 2021; Lou et al., 2021; Zhang et al., 2021; Chang et al., 2022; Rothwell et al., 2022). Here, we tested 30 gene expression-based scores in the seven cohorts used in this study (Figure 7; Supplementary Table 12), and the results were compared with our GES7. Only two scores, which include the GES7, were the robust for overall survival prediction, highlighting its potential clinical use. Possible explanations can be raised up for this finding. First, here we tested our GES7 in cohorts from different centers around the world, which means that our score is widely applicable independent of ethnicity. Secondly, we performed an independent analysis in seven GC datasets, which describes our score as robust. Thirdly, we used genes that predict deaths and relapse events by themselves. In addition, we found an improvement in the TNM risk stratification. The TNM system has been criticized for its poor stratification ability (Dikken et al., 2012; Marrelli et al., 2012; Yoon et al., 2012; Shu et al., 2017). Although in the most recent update (8th edition), some modifications were made, such as the division of the pN3 stage into pN3a and pN3b (Amin et al., 2017; Lu et al., 2017; Ji et al., 2018), one study concluded that this latest version has similarities to the previous one in terms of its predictive capabilities (Abdel-Rahman, 2017). Then, the inclusion of our GES7 could be a multigenic option to improve GC prognosis. Finally, the GES7 signature is associated with sensitivity to specific drugs. This last result still needs to be validated but opens the possibility of applying potential drugs for GES7^high^ patients.

The discovery process for genes associated with prognosis and for building scores used in this study relies on classic statistics. Nowadays, more sophisticated modeling approach, like machine learning algorithms, can be used to capture and model complex non-linear relationships in the data, which may be challenging for classic statistical models (Zhang et al., 2023). However, the implementation of these algorithms can sometimes introduce challenges in interpretability and may not always guarantee better performance, especially in situations with limited sample sizes or noisy data. The simplicity of our index is advantageous, as it enables easier clinical translation and adoption in routine practice. On the other hand, we recognize limitations to our study, such as the use of previously published data and reliance on self-reported participant information. Furthermore, the inclusion of a prospective validation cohort will strengthen the findings. Also, it will be important to explore more affordable methods for measuring GES7, such as QPCR, to facilitate its future use in routine clinical practice.

Taken together, our findings provide compelling evidence for the relevance of GES7 as a prognostic marker in gastric cancer, and we hope that our results will inspire further research in this area.

## Data Availability

Publicly available datasets were analyzed in this study. This data can be found here: The Cancer Genome Atlas (TCGA) was obtained from http://firebrowse.org/ (TCGA). The microarray data from Asian Cancer Research Group (ACRG), Yonsei University Severance Hospital (YUSH), Korea University Guro Hospital (KUGH), National Cancer Centre of Singapore (NCCS), Kosin University College of Medicine (KUCM), and The University of Texas MD Anderson Cancer Center (MDACC), was obtained from https://www.ncbi.nlm.nih.gov/geo/.
