# Supplementary figures and images for: Establishment of a 7-gene expression panel to improve the prognosis classification of gastric cancer patients
Source: Front Genet. 2023 Sep 12;14:1206609. doi: 10.3389/fgene.2023.1206609 (PMC10522918; doi:10.3389/fgene.2023.1206609)

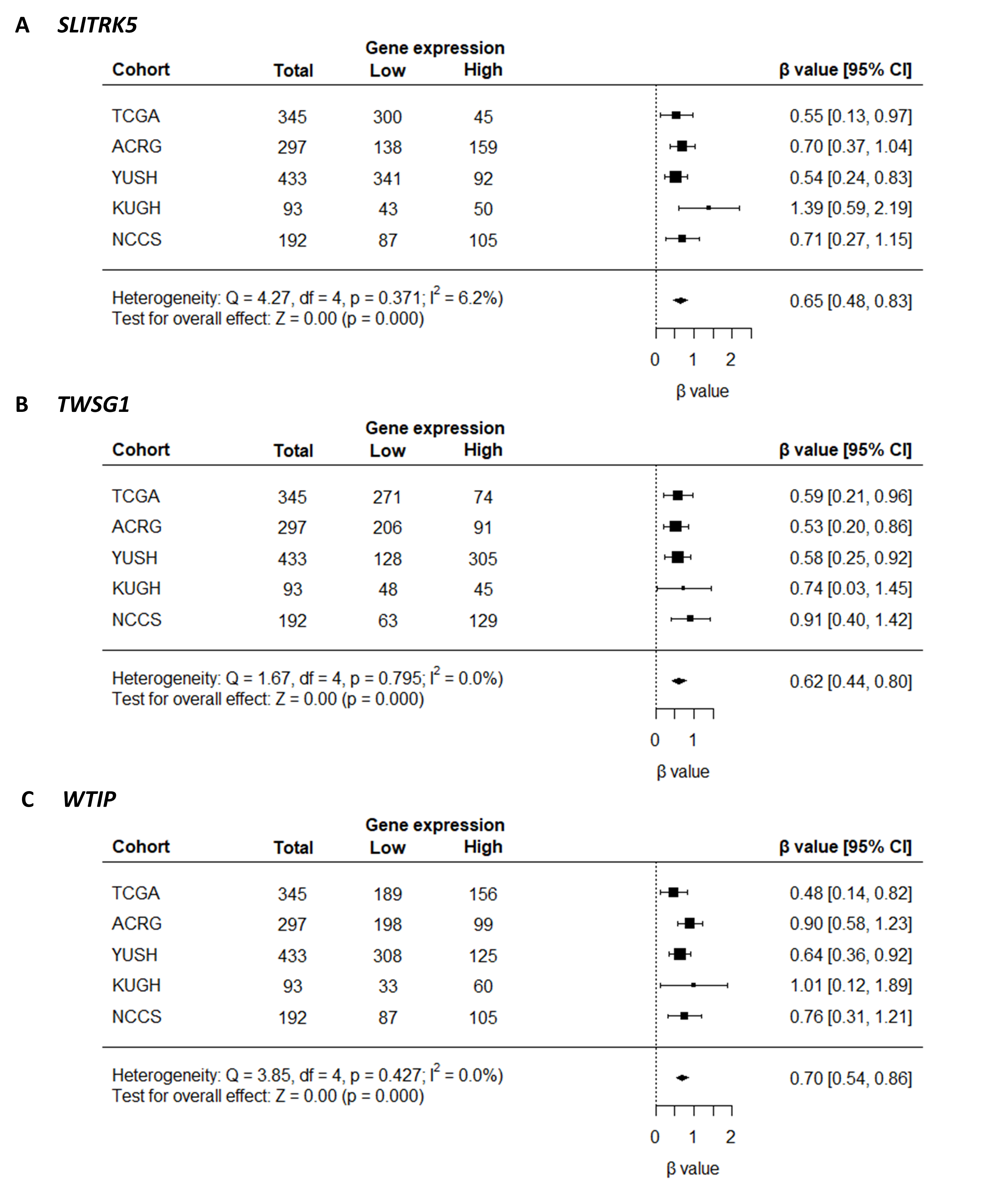

Supplement: Supplementary file 1 [file Image3.TIFF]

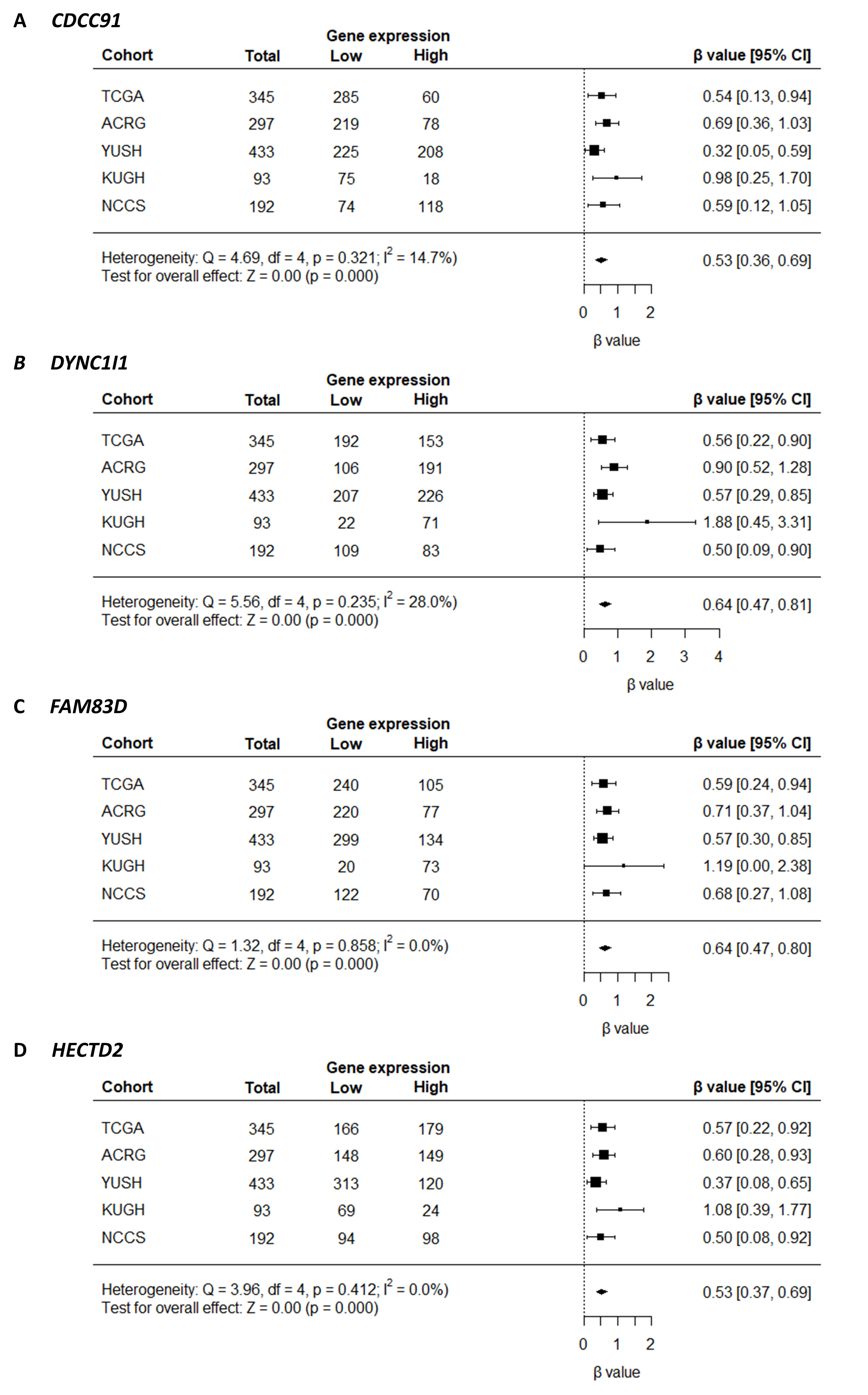

Supplement: Supplementary file 2 [file Image1.TIFF]

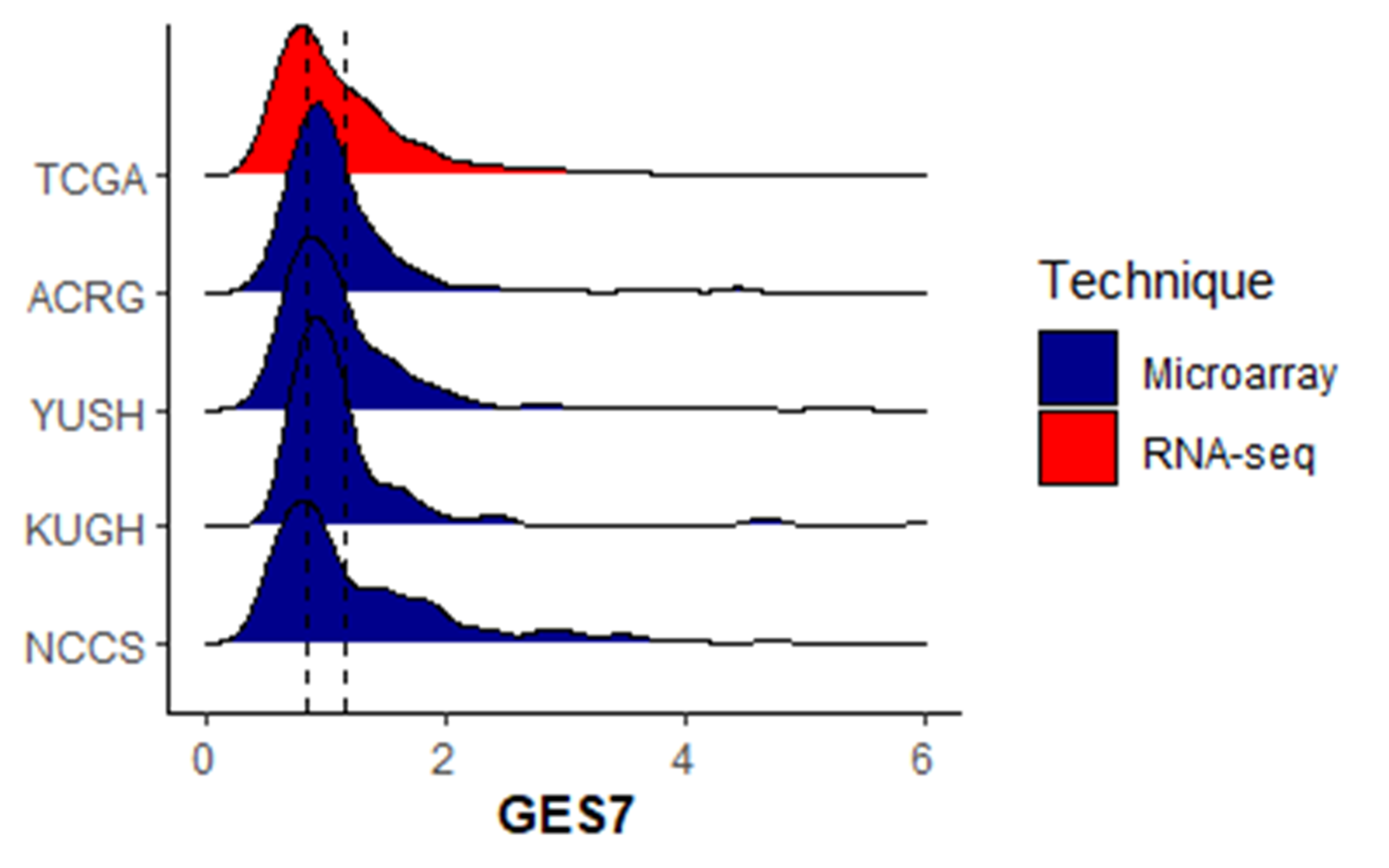

Supplement: Supplementary file 3 [file Image9.TIFF]

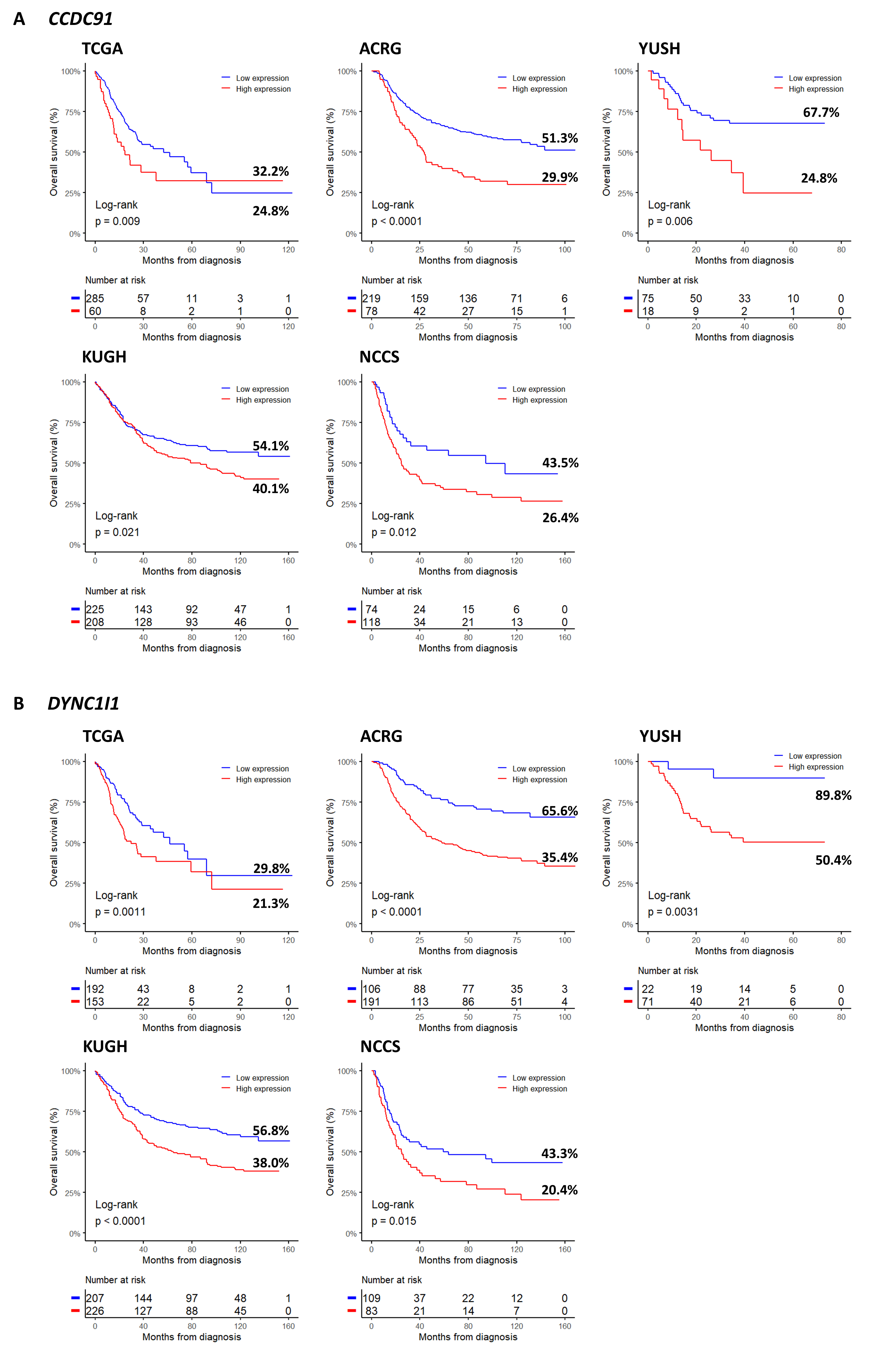

Supplement: Supplementary file 4 [file Image5.TIFF]

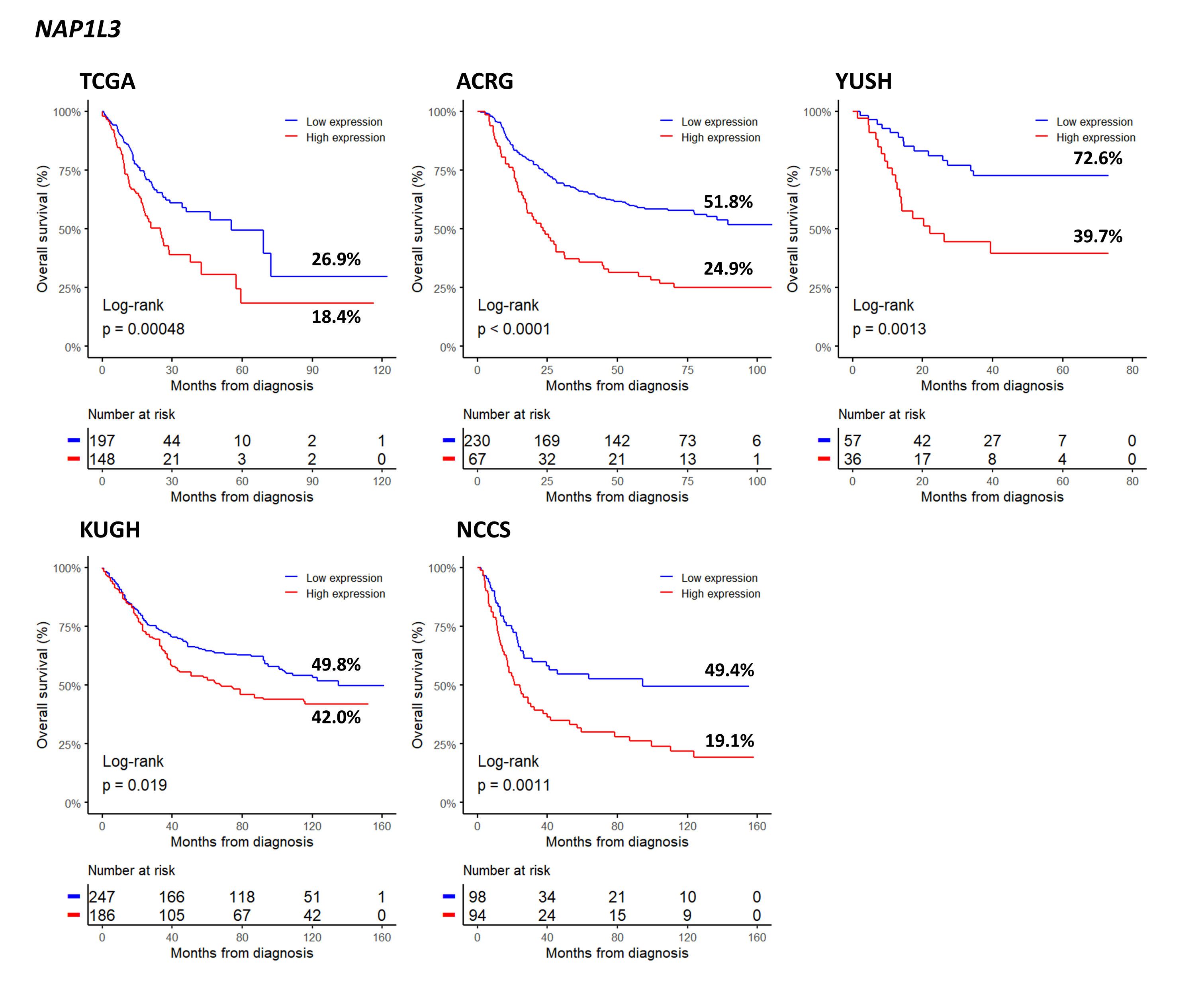

Supplement: Supplementary file 5 [file Image8.TIFF]

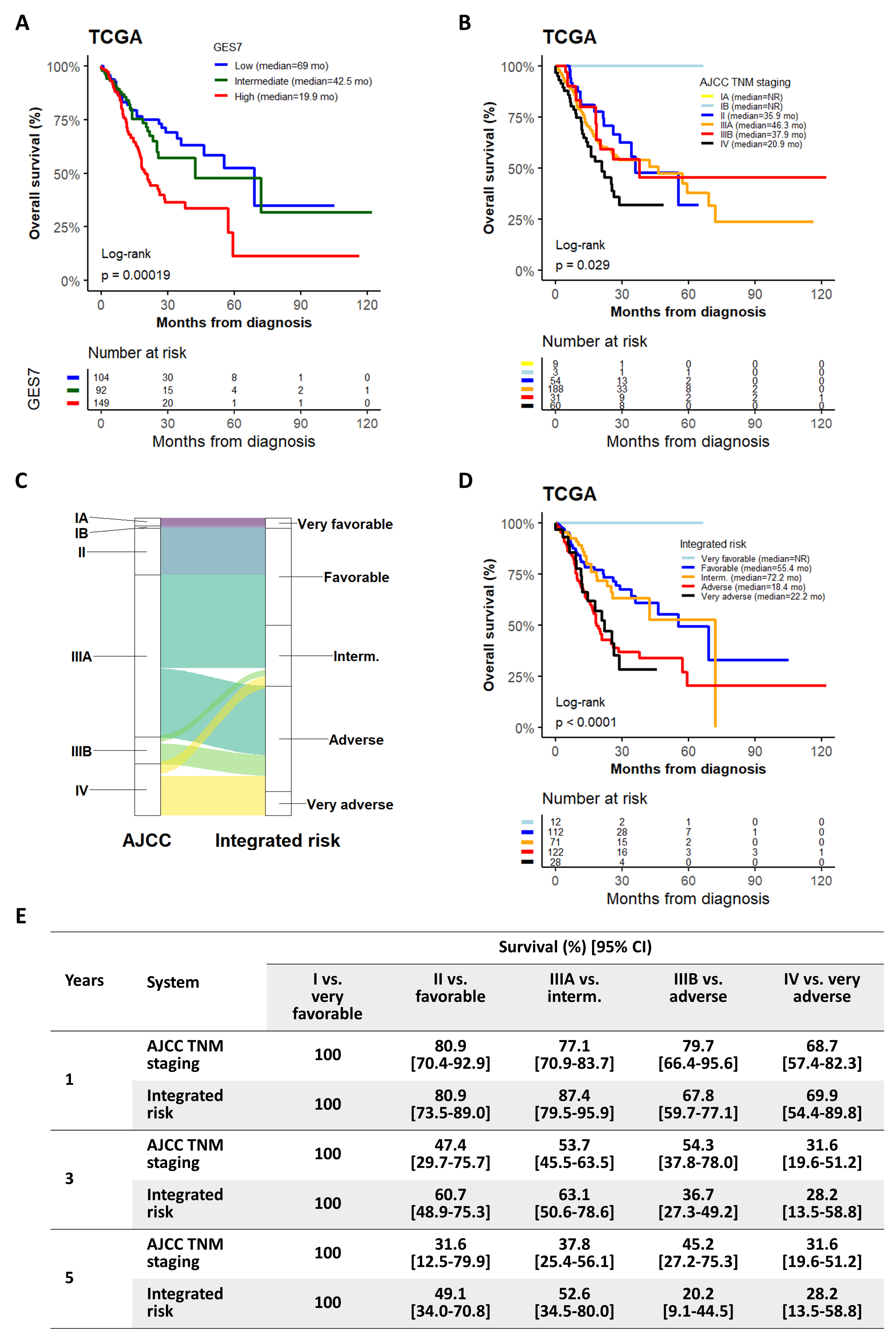

Supplement: Supplementary file 6 [file Image11.TIFF]

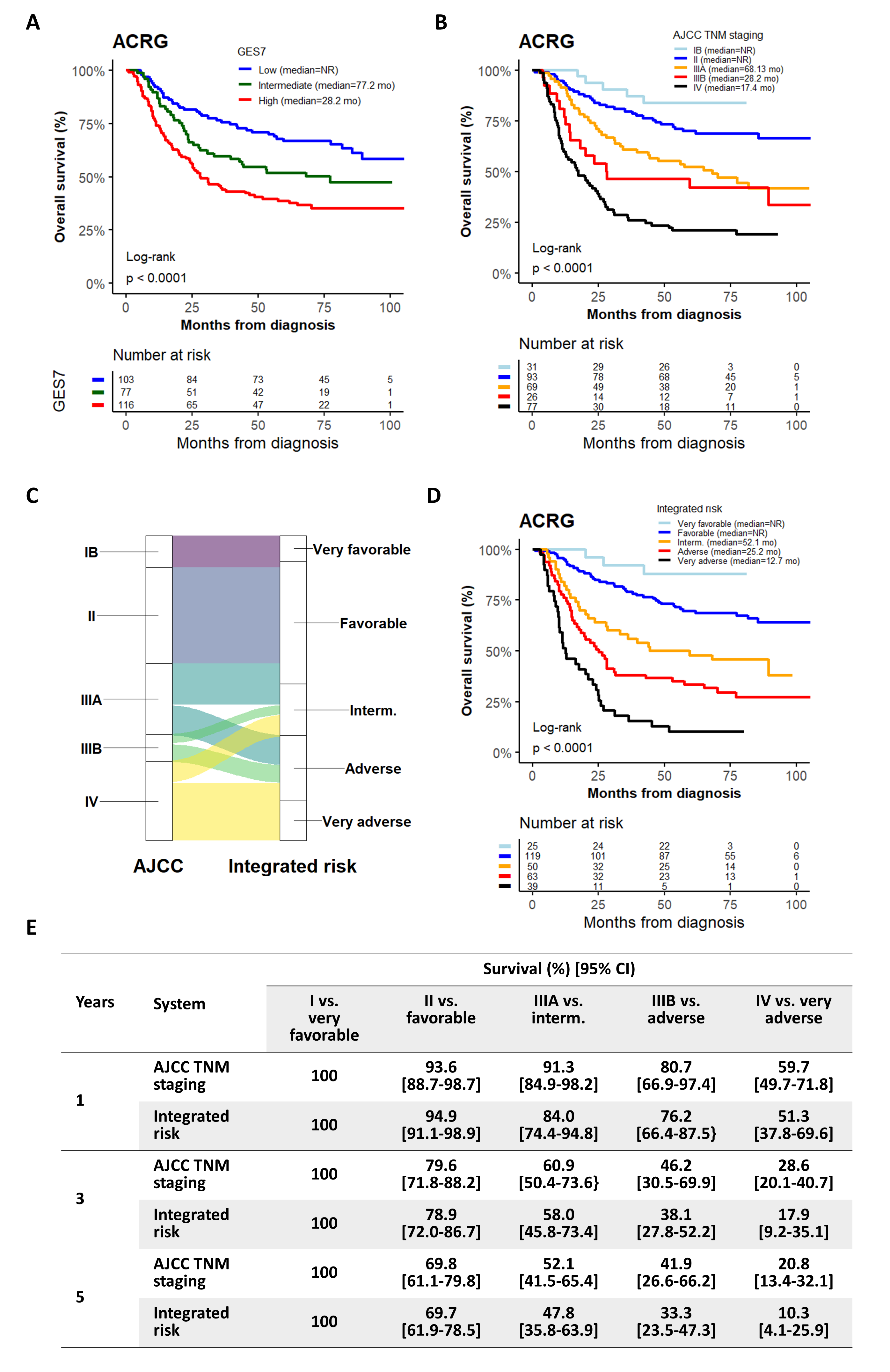

Supplement: Supplementary file 8 [file Image10.TIFF]

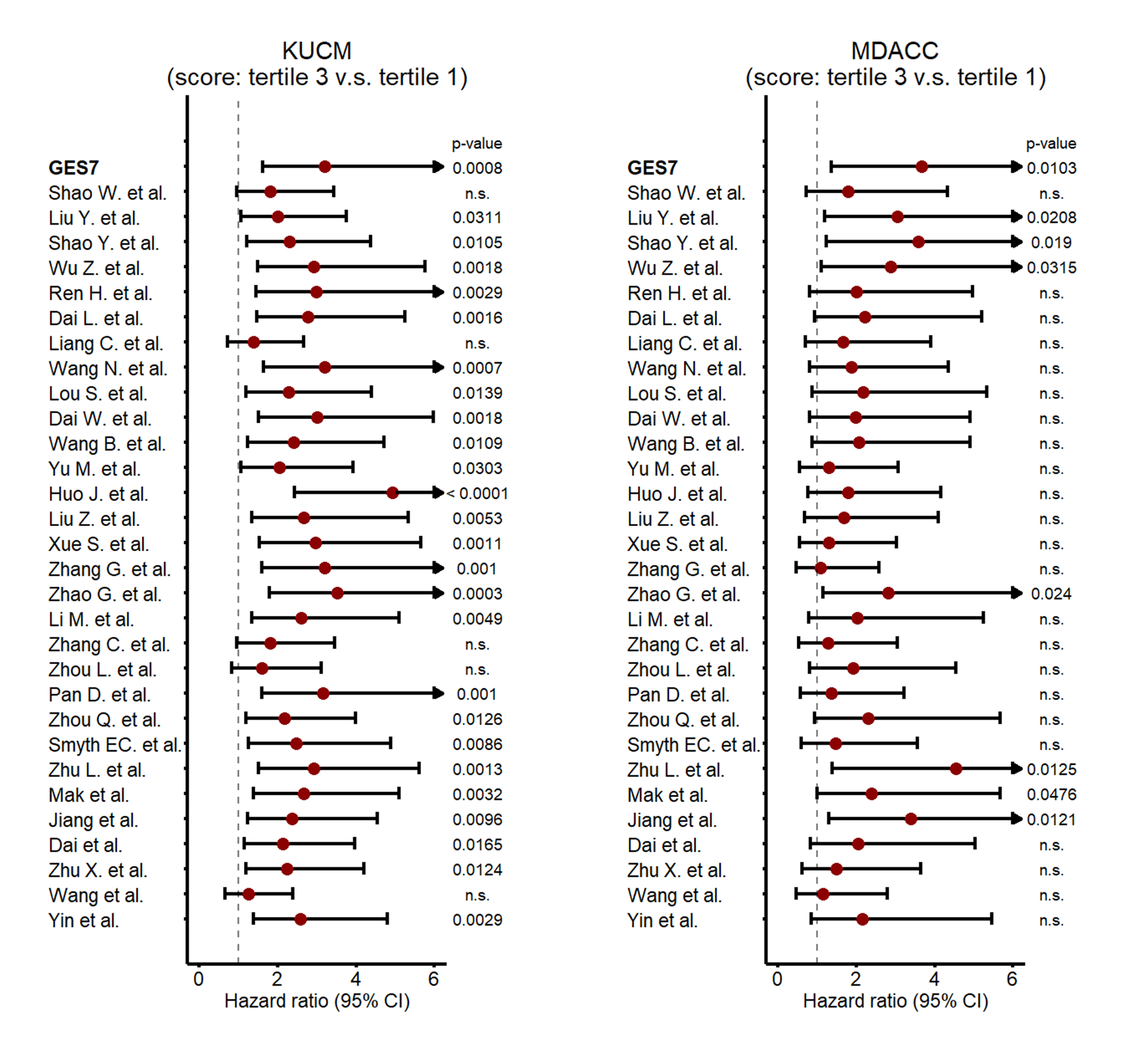

Supplement: Supplementary file 9 [file Image12.TIFF]

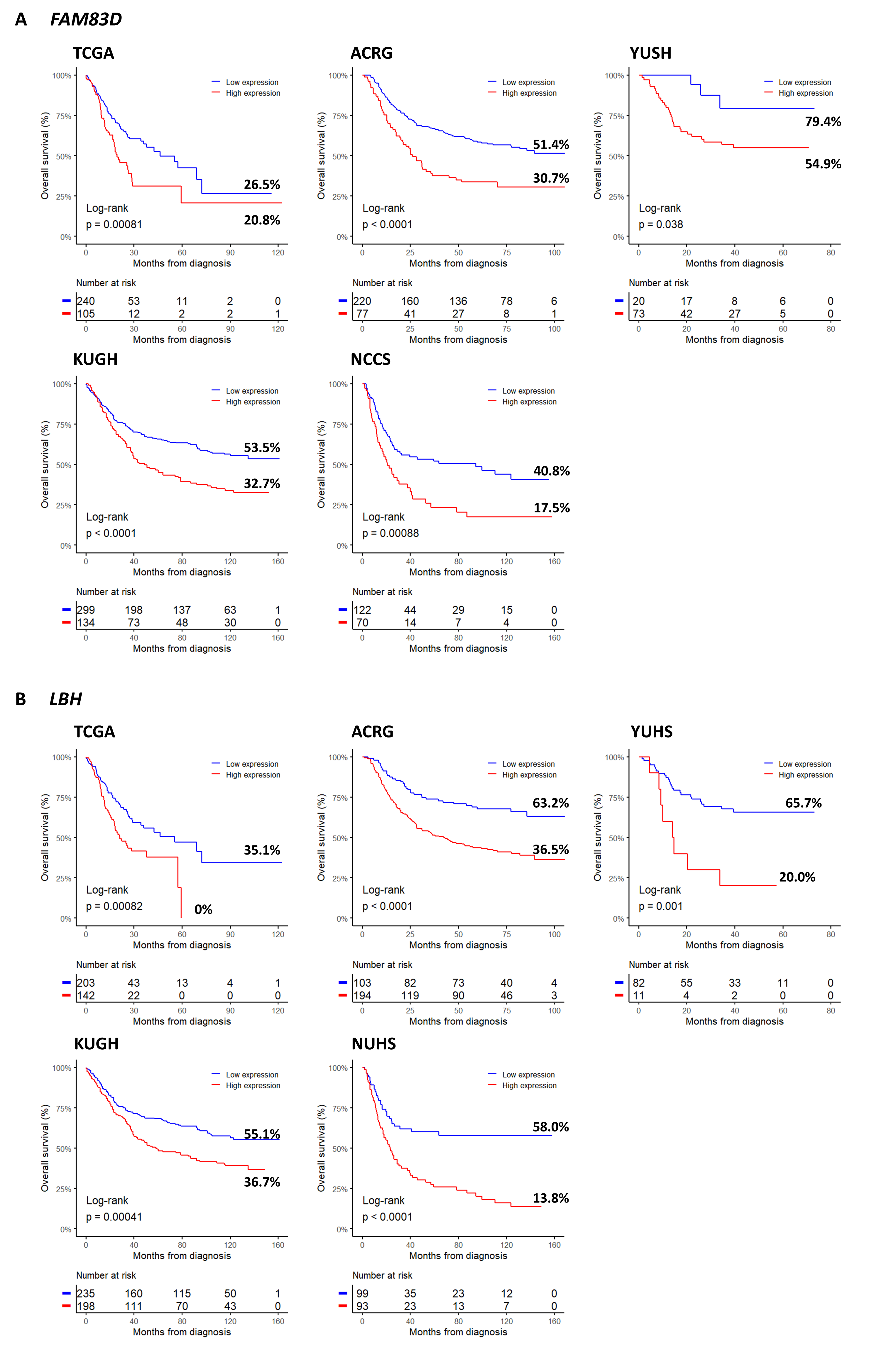

Supplement: Supplementary file 10 [file Image6.TIFF]

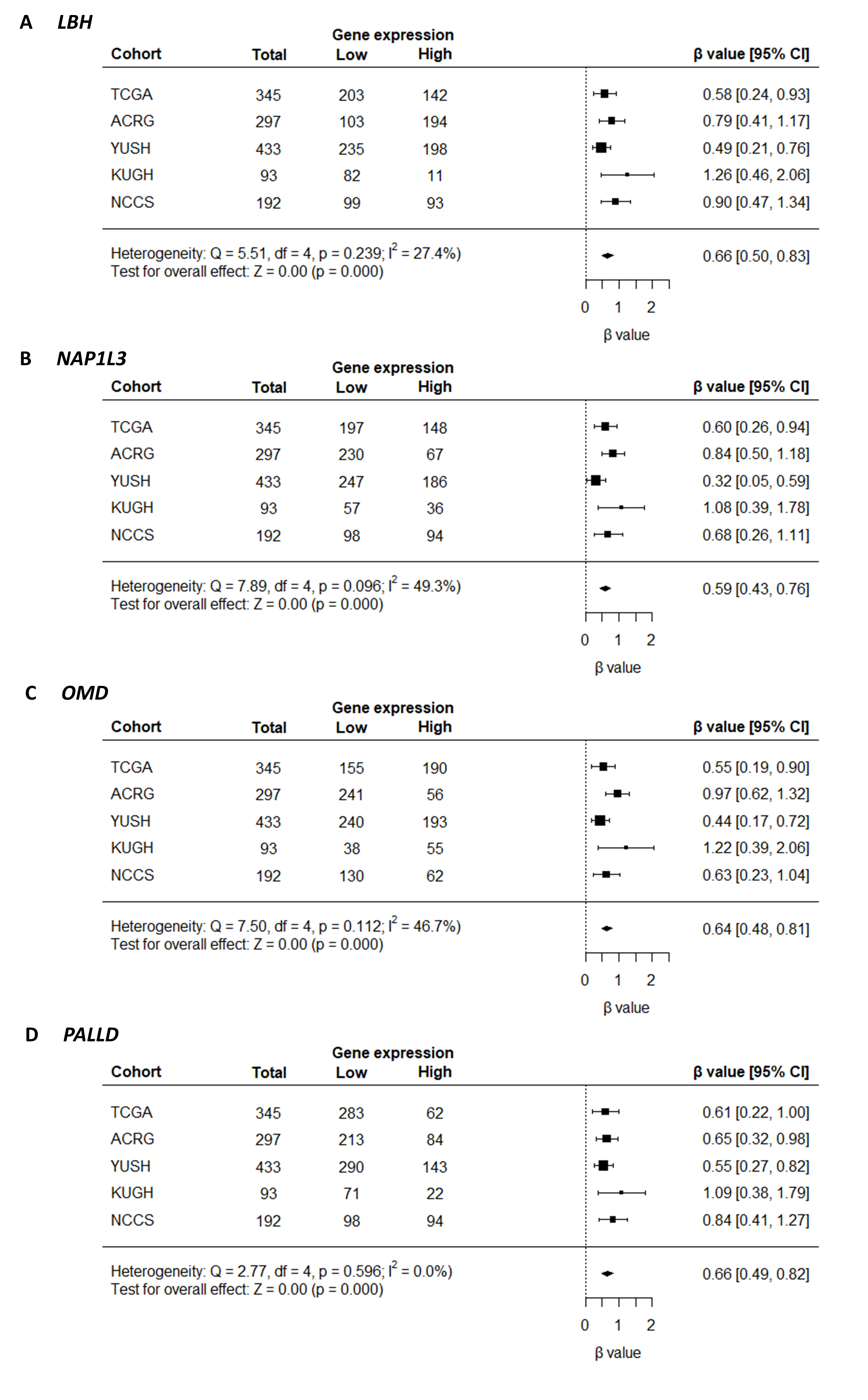

Supplement: Supplementary file 11 [file Image2.TIFF]

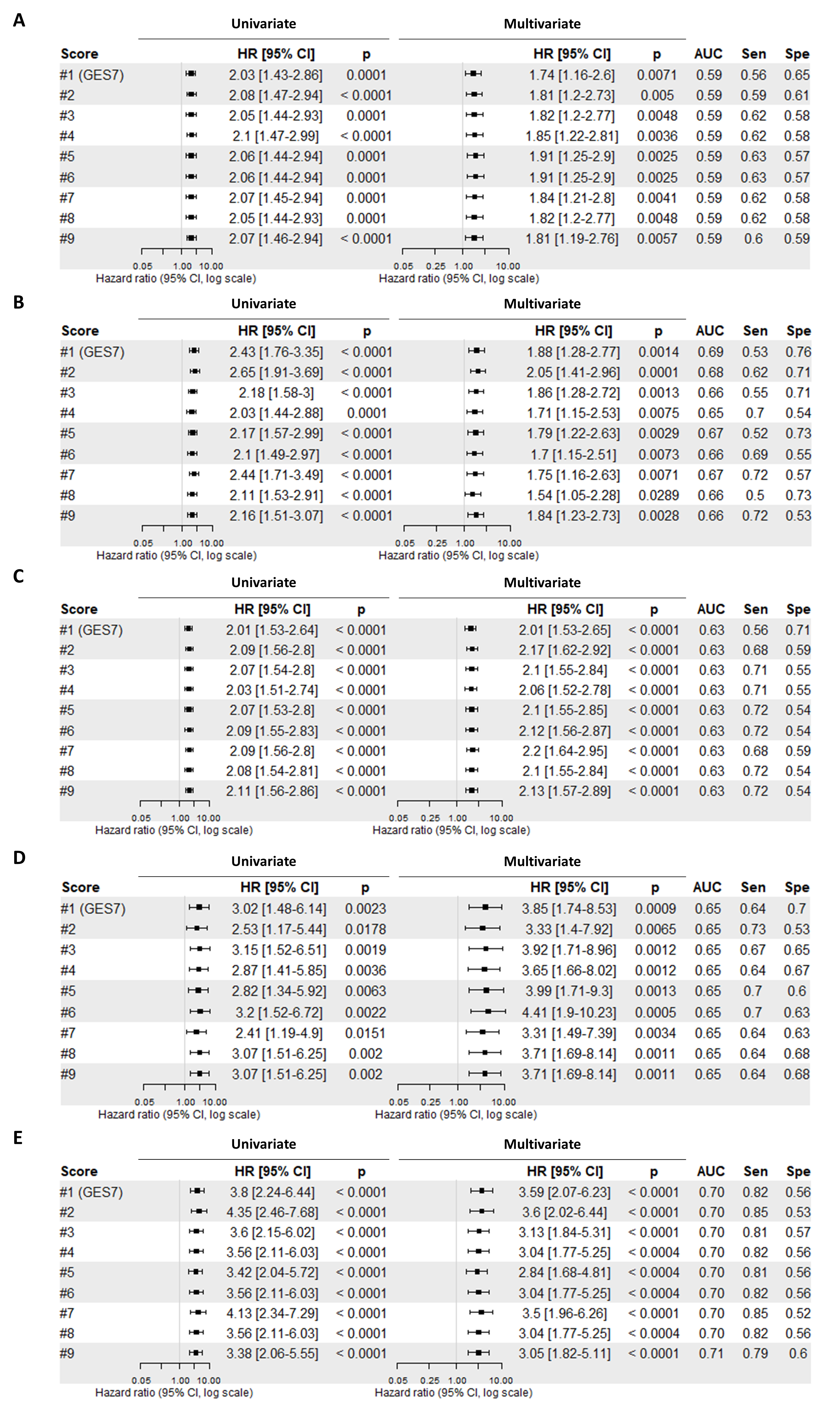

Supplement: Supplementary file 12 [file Image4.TIFF]

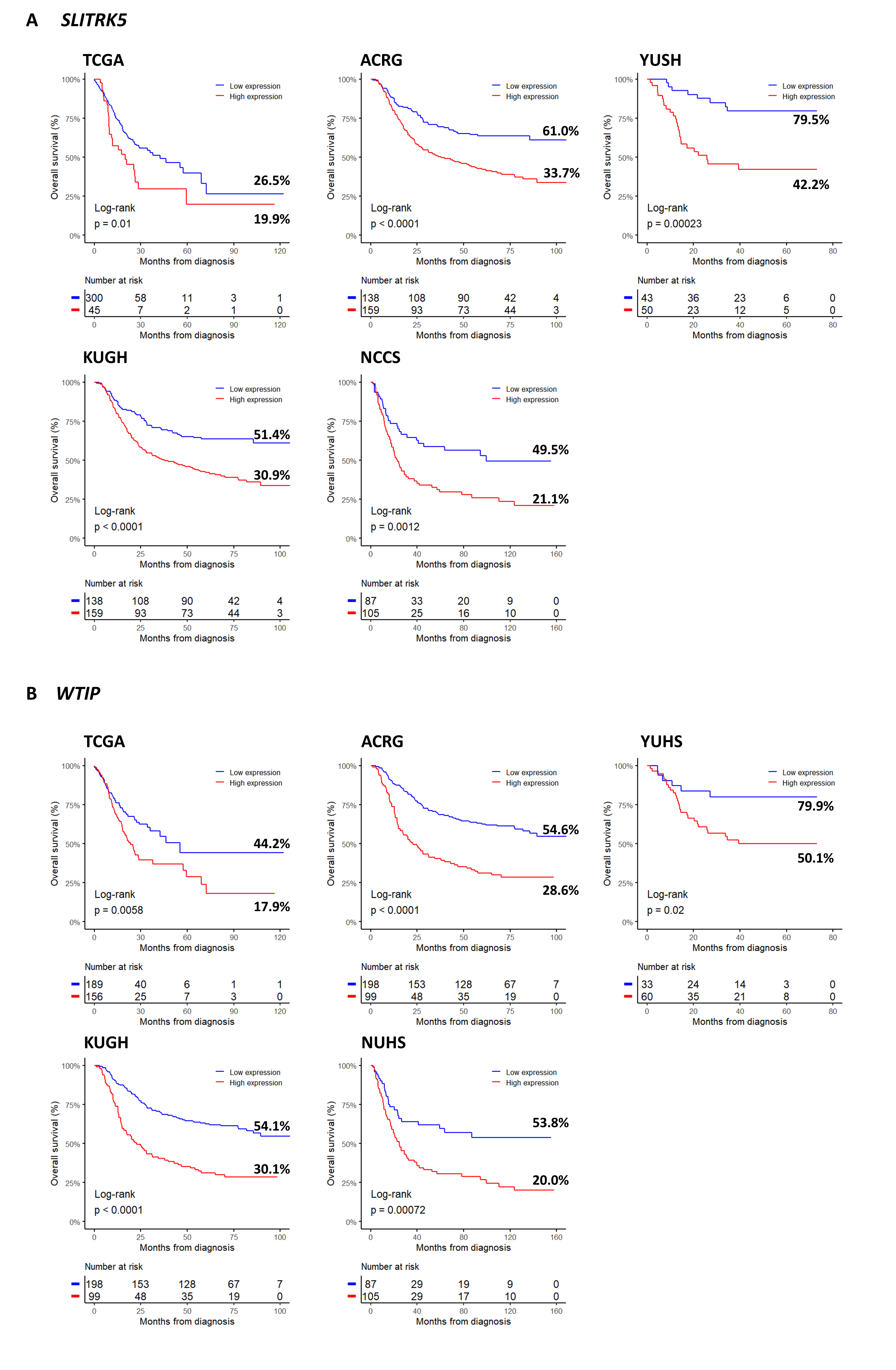

Supplement: Supplementary file 13 [file Image7.TIFF]
